# A Disturbed Siderophore Transport Inhibits Myxobacterial Predation

**DOI:** 10.3390/cells11233718

**Published:** 2022-11-22

**Authors:** Yijie Dong, Honghong Dong, Zengwei Feng, Xing Wang, Qing Yao, Honghui Zhu

**Affiliations:** 1 Key Laboratory of Agricultural Microbiomics and Precision Application, Guangdong Provincial Key Laboratory of Microbial Culture Collection and Application, Key Laboratory of Agricultural Microbiome (MARA), State Key Laboratory of Applied Microbiology Southern China, Guangdong Microbial Culture Collection Center (GDMCC), Institute of Microbiology, Guangdong Academy of Sciences, Guangzhou 510070, China; dongyj@gdim.cn (Y.D.); donghh@gdim.cn (H.D.); fengzw@gdim.cn (Z.F.); 20212118036@stu.kust.edu.cn (X.W.); 2 College of Horticulture, Key Laboratory of Microbial Signals and Disease Control, South China Agricultural University, Guangzhou 510642, China; yaoqscau@scau.edu.cn

**Keywords:** siderophores, ABC transporter, myxobacterial predation, outer membrane vesicles

## Abstract

Background: Understanding the intrinsic mechanisms of bacterial competition is a fundamental question. Iron is an essential trace nutrient that bacteria compete for. The most prevalent manner for iron scavenging is through the secretion of siderophores. Although tremendous efforts have focused on elucidating the molecular mechanisms of siderophores biosynthesis, export, uptake, and regulation of siderophores, the ecological aspects of siderophore-mediated competition are not well understood. Methods: We performed predation and bacterial competition assays to investigate the function of siderophore transport on myxobacterial predation. Results: Deletion of *msuB*, which encodes an iron chelate uptake ABC transporter family permease subunit, led to a reduction in myxobacterial predation and intracellular iron, but iron deficiency was not the predominant reason for the decrease in the predation ability of the ∆*msuB* mutant. We further confirmed that obstruction of siderophore transport decreased myxobacterial predation by investigating the function of a non-ribosomal peptide synthetase for siderophore biosynthesis, a TonB-dependent receptor, and a siderophore binding protein in *M. xanthus*. Our results showed that the obstruction of siderophores transport decreased myxobacterial predation ability through the downregulation of lytic enzyme genes, especially outer membrane vesicle (OMV)-specific proteins. Conclusions: This work provides insight into the mechanism of siderophore-mediated competition in myxobacteria.

## 1. Introduction

Bacteria rarely exist in isolation but instead more constantly inhabit complex microbial communities [1]. Microbial communities colonize different niches and exhibit different scales from hundreds to billions of cells [2]. The interspecies interactions between microbial communities can produce various outcomes ranging from cooperation to competition or commensalism. However, compared with cooperative or commensal interactions, competitive interactions appear to be more common [3,4]. Different bacterial species compete for scarce nutrients and limited space, and this competition serves as an important part of microbial life and is a pivotal evolutionary driver for various attack and defense mechanisms [5].

Iron is essential for all forms of life by functioning as cofactors involved in many biological processes. Meanwhile, it is also one of the essential nutrients that bacteria struggle for [6]. Bacteria capture iron by secreting siderophores. Siderophores are kinds of low molecular weight iron-chelating compounds, the role of which is thought to be in facilitating iron scavenging, transport, and uptake [7]. Generally, the outer-membrane receptors (OMRs), periplasmic binding proteins (PBPs), TonB complex, and ABC-type transporters are involved in the transport of iron–siderophore complexes in Gram-negative bacteria [8]. In contrast, due to the absence of an outer membrane or periplasmic zone, the iron–siderophore complexes are directly perceived by the PBPs’ receptors and then transported with the ABC transporter into the cytoplasm in Gram-positive bacteria [9]. Bacteria secrete siderophores with the dual effects of nutrient acquisition and limiting the iron available to its competitors. Bacteria’s fine-tuned production of siderophores may be an effective strategy to lock iron away from competing species. Under iron-limiting conditions, *Vibrio fischeri* ES114 can competitively inhibit *V. harveyi* by producing aerobactin, which does not produce and uptake aerobactin. When *V. fischeri* ES114 is unable to produce aerobactin, it will lose its competitive advantage over iron [10]. *Pseudomonas aeruginosa* plastically adjusts the production of pyoverdine in response to the level of iron competition imposed by *Burkholderia cenocepacia* [11]. Thus, siderophores might play an important role in mediating interactions between interspecific or intraspecific cells.

*Myxococcus xanthus* is a predatory bacterium that can prey on a broad range of bacteria and fungi and plays an important role in shaping microbial communities [12,13,14,15,16,17]. *M. xanthus* can prey collaboratively on other microbial cells in a wolf-pack pattern [18]. Although *P. aeruginosa* uses the Type 6 secretion system for killing the competitor in a contact-dependent manner, some studies have found that *M. xanthus* can prey on *P. aeruginosa* [12,19]. Myxochelin A, a catecholate siderophore, was originally isolated from a culture broth of myxobacterium *Angiococcus disciformis* An d30 [20]. The biosynthetic gene cluster of myxochelin-type siderophores was identified in *Stigmatella aurantiaca* Sg a15 and *Sorangium cellulosum* So ce56, and disruption of the myxochelin biosynthesis pathway leads to severe growth defects in myxobacteria under iron-limiting conditions [21,22]. A previous study demonstrated that *M. xanthus* can produce myxochelin A and B for iron acquisition in order to maintain intracellular iron homeostasis [23]. LC-MS analysis demonstrated that myxochelin is ubiquitous in *M. xanthus*, suggesting that it provides a significant fitness benefit to *M. xanthus* [23,24]. Alongside iron acquisition, the purified myxochelins also have antibacterial activity and antiproliferative activity by inhibiting human 5-lipoxygenase [25,26]. A recent study showed that *M. xanthus* increased the production of myxochelin and led *Streptomyces coelicolor* to experience iron-restricted conditions during coculture with *S. coelicolor* [27], indicating that siderophores derived from *M. xanthus* might have a stronger affinity to iron under microbial competition.

Bacterial outer membrane vesicles (OMVs) are spherical-, bilayered-, membranous structures (20–400 nm diameter) derived from the outer membrane of Gram-negative bacteria [28]. OMVs serve as a unique bacterial secretion pathway and are involved in bacterial interactions in microbial communities [29]. *M. xanthus* can produce prolific OMVs, which might be responsible for delivering a complex mixture of metabolites and enzymes to the prey [17]. A previous study has indicated that the OMVs generated by *M. xanthus* are able to kill *Escherichia coli* and a *Pseudomonas* sp. strain by fusing with their outer membranes and delivering cargo into the prey cytoplasm/periplasm [30]. Berleman and collaborators analyzed the secondary metabolite profile of myxobacterial OMVs using reversed phase liquid chromatography mass spectrometry (RP LC-MS) and confirmed that myxochelin A and myxochelin B were present in OMVs [31]. *P. aeruginosa* can enrich the highly hydrophobic iron chelator Pseudomonas quinolone signal (PQS) into OMVs for iron uptake [29]. However, the potential connection between siderophores and OMVs during myxobacterial competition has hitherto been unclear.

Here, we provide the first description of the *msuABCD* gene cluster encoding the ferric siderophore uptake ABC transporter and explore its functions in myxobacterial predation on *P. aeruginosa*. Our results showed that disruption of the *msuB* gene in *M. xanthus* led to a reduction in the predation ability and intracellular iron level, suggesting that disordered iron homeostasis may affect myxobacterial predation. We further confirmed that the interruption of siderophore transport, rather than iron deficiency, was the major reason for decreasing the predation ability of ∆*msuB* mutant. Next, our results demonstrated that the siderophores are involved in myxobacterial predation by affecting the expression of the outer membrane vesicle proteins.

## 2. Materials and Methods

### 2.1. Bacterial Strains and Growth Media

The strains and plasmids used in this study are listed in Supplementary Table S1. All *M. xanthus* strains are derived from DK1622 [32]. In-frame deletion mutants were constructed using the pBJ113 plasmid [33,34]. The pSWU30 plasmid for ectopic expression of the corresponding genes was transformed into the mutant and integrated into the chromosome at the *attB* site in *M. xanthus* [35]. Complementation of *msuB* deletion (∆*msuB/msuB*) was achieved by expressing *msuB* under the control of its own promoter. All plasmids were verified by sequencing. The deletion mutants were confirmed by PCR. *M. xanthus* strains were grown at 30 °C in a CTT medium (1% casitone, 10 mM Tris-HCl (pH 7.6), 1 mM KPO_4_ pH 7.6, 8 mM MgSO_4_) or on CTT 1.5% agar plates supplemented with kanamycin (40 µg mL^−1^) or tetracycline (10 µg mL^−1^) when required. *E. coli* strains were grown in LB broth. Plasmids were propagated in *E. coli* TOP10. *P. aeruginosa* PAO1 was grown in LB broth or on LB agar plates supplemented with tetracycline (20 µg mL^−1^).

### 2.2. Predation Assay on Agar Plates

A predation assay was performed on TPM plates (10 mM Tris-HCl (pH 7.6), 1 mM KPO_4_ pH 7.6, 8 mM MgSO_4_, 1.5% agar) using the colony-induced predation method as described [36,37]. Overnight cultures of *P. aeruginosa* PAO1 were resuspended in an LB medium and cultured to the mid-exponential phase. The cells were collected, washed twice with MMC buffer (10 mM MOPS pH 7.6, 4 mM MgSO_4_, 2 mM CaCl_2_), and resuspended in MMC to OD_550 nm_ = 50. In total, 100 µL of PAO1 cells was inoculated on TPM (1.5% agar) plates and allowed to dry. For monitoring the effect of different iron concentrations on myxobacterial predation (200 µM, 500 µM and 800 µM FeCl_3_) was added to the TPM (1.5% agar) plates. The *M. xanthus* strains were grown in CTT for 24 h, and the cells were in the exponential growth phases (OD_550 nm_ ~0.6). The cells were collected by centrifugation and washed twice with MMC buffer and resuspended in MMC buffer to OD_550 nm_ = 10 (1 × 10^10^ cells mL^−1^). Next, 3 µL of the cells was spotted at a 2 mm distance from the PAO1 colony. The plates were cultured at 30 °C for the indicated time. The lysed area of *P. aeruginosa* was observed with a stereomicroscope (Olympus SZX10, Olympus Corporation, Tokyo, Japan).

### 2.3. Bacterial Competition Assay

The competition assay was performed as previously described with minor modifications [38]. The cells of *P. aeruginosa* PAO1 at the mid-exponential phase were harvested and resuspended in MMC buffer to OD_550 nm_ = 10. *M. xanthus* was grown in CTT for 24 h and the cells were adjusted to OD_550 nm_ = 10. Cells of different *M. xanthus* strains and PAO1 were mixed in a 1:1 ratio, and 100 µL of the mixture was spotted on dry TPM (1.5% agar) plates for coculture at 30 °C. As a control, 50 µL of PAO1 (OD_550 nm_ = 10) was used. After 24 h, the cocultured *M. xanthus* strains and PAO1 were harvested, and the cells were resuspended in 1 mL of MMC buffer. Subsequently, the cellular suspension was serially diluted in MMC buffer, and 3 µL of the suspensions was spotted on LB agar plates supplemented with tetracycline (20 µg mL^−1^). The growth of *M. xanthus* was inhibited on LB agar plates containing tetracycline. Colonies, which represented the survival of *P. aeruginosa* PAO1, were photographed after 24 h of incubation at 30 °C. At least three biological replicates were performed. To assess the effect of different iron levels on myxobacterial predation, 1 mM, 2 mM, and 4 mM FeCl_3,_ or 50 µM, 100 µM, and 200 µM bathophenanthroline disulfonate (BPS) was added to the CTT medium.

### 2.4. Iron Content Determination

Cellular iron concentrations were detected according to the BPS-based colorimetric method as described previously [39,40]. Different *M. xanthus* strains were grown in a CTT medium for 24 h. The cells were harvested and resuspended in 500 µL of 3% nitric acid, and completely lysed by boiling for 2 h. Next, 400 µL of the supernatants was then mixed with 160 µL of 38 mg mL^−1^ sodium ascorbate, 320 µL of 3 mM BPS, and 126 µL of 4 M sodium acetate. After incubation at room temperature for 10 min, the OD_535 nm_ of the BPS-Fe complex was measured by Multiskan SkyHigh (ThermoFisher Scientific). OD_680 nm_ was also detected as the nonspecific absorbance. The iron content was calculated via the formula: (OD_535 nm_—OD_680 nm_)/cell number, and displayed in arbitrary units (A.U.). The data are presented as means ± standard error (SE).

### 2.5. Chromeazurol S Overlay (O-CAS) Assay

The chromeazurol S overlay (O-CAS) assay was performed for detecting the content of siderophores as previously described, with some modifications [41]. Briefly, CAS Blue Dye was made by combining the following: 50 mL of Solution 1 (60.5 mg chromeazurol S dissolved in 50 mL deionized H_2_O), 10 mL of Solution 2 (16.2 mg ferric chloride hexahydrate dissolved in 100 mL 10 mM hydrochloric acid), 40 mL of Solution 3 (72.9 mg hexadecyl trimethyl ammonium bromide (HDTMA) dissolved in 40 mL of deionized H_2_O), and 900 mL of deionized H_2_O. The agarose was added as a solidifying agent. To make the CAS overlay, 1.0 g of an agarose solution was added to 100 mL of CAS Blue Dye and heated to melt the agarose. *M. xanthus* strains were grown in CTT for 24 h. The cells were harvested and resuspended in 1 mL of MMC buffer and adjusted to OD_550 nm_ = 10, and 3 µL of the suspension was spotted on a CAA plate (5 g∙L^−1^ low-iron CAA (Difco), 1.46 g∙L^−1^ K_2_HPO_4_∙3H_2_O, 0.25 g∙L^−1^ MgSO_4_∙7H_2_O and 15 g∙L^−1^ agar) for 3 days. Next, 15 mL of the resulting O-CAS solution was overlaid onto the CAA plates. The plates were photographed after 24 h of incubation at 30 °C. At least three biological replicates were performed.

### 2.6. Transcriptome Analysis

*M. xanthus* DK1622 and ∆*msuB* mutants were grown in a CTT medium for 24 h at 30 °C. Total RNA was isolated and then the mRNA was purified using probes to remove rRNA for mRNA-seq library construction. The libraries were sequenced on an Illumina Novaseq platform to generate paired-end reads of 150 bp in length (Novogene Bioinformatics Technology Co., Ltd.). Clean reads were trimmed to remove adapter sequences and mapped to the DK1622 genome using Bowtie2-2.2.3. Differentially expressed genes (DEGs) were calculated with the cut-off of a fold change of >0 or a fold change of <0, and *P*_adj_ < 0.05 using DESeq2. For the Kyoto Encyclopedia of Genes and Genomes (KEGG) analysis, DEGs with a fold change of >0 or a fold change of <0 (*P*_adj_ < 0.01) were included and the statistical enrichment of differentially expressed genes in the KEGG pathways were analyzed using KOBAS software.

### 2.7. RNA Extraction and Quantitative Real-Time PCR (qRT-PCR)

*M. xanthus* was grown in CTT for 24 h at 30 °C. The samples were harvested by centrifuging a 12,000× *g* at 4 °C, flash-frozen in liquid nitrogen, and stored at −80 °C. Total RNA was extracted using a HiPure Bacterial RNA Kit (Guangzhou Magen Biotechnology Co., Ltd., Guangzhou, China). Total RNA (1 µg) was used to synthesize cDNA using the TransScript Uni One-Step gDNA Removal and cDNA Synthesis SuperMix (TransGenBiotech, Beijing, China), according to the manufacturer’s instructions. Quantitative RT-PCR (qRT-PCR) was performed in 20 µL reaction mixtures using SYBR Green PCR master mix (TransGenBiotech), 2.0 µL of cDNA product, and 0.2 µM of each primer in a QuantStudio 6 Flex system (Applied Biosystems, Waltham, MA, USA). The relative expression of the target genes was calculated using the 2^−ΔΔCt^ method [42]. The gapA (*MXAN_2815*) gene was used as the internal standard. The data are presented as means ± standard error (SE).

### 2.8. Isolation of OMVs

*M. xanthus* DK1622 and ∆*msuB* mutants were grown in a CTT medium for 24 h at 30 °C. In total, 100 mL of the liquid culture was vortexed for 30 s and then centrifuged for 10 min at 5000× *g* to obtain a supernatant containing OMVs. Subsequently, the supernatant was sequentially passed through 0.45 µm and 0.22 µm filters for removing the cellular debris. The cell-free liquid was centrifuged at 150,000× *g* for 1 h at 4 °C. The resulting pellet was resuspended in 500 µL of PBS to obtain the OMVs.

### 2.9. Data Availability

All data supporting this study are available within the article and the supplemental information files. Raw RNA-seq data for this study were deposited at the National Center for Biotechnology Information website SRA under PRJNA849267.

## 3. Results

### 3.1. Loss of msuB Function Affects Myxobacterial Predation

**Figure 1:**
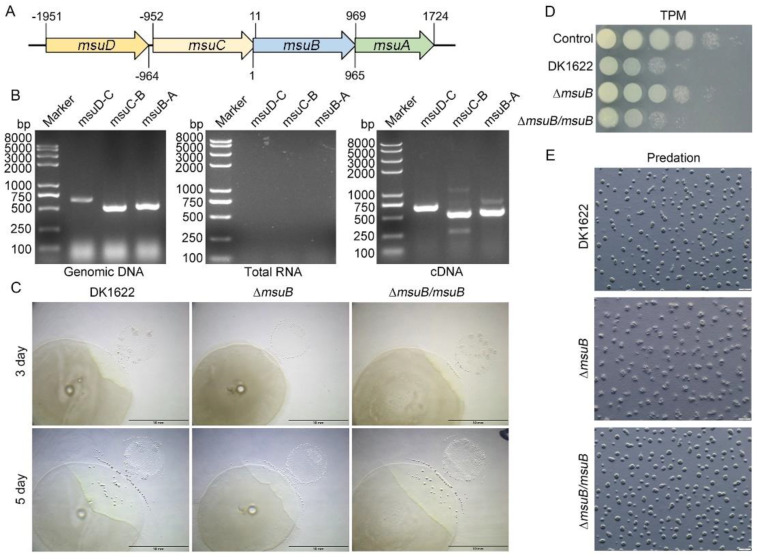
MsuB plays an important role in myxobacterial predation. (**A**) *msuABCD* locus. Start and stop codons are indicated; +1 indicates the first nucleotide in the *msuB* start codon. (**B**) Cotranscriptional analysis of the *msuABCD* cluster by reverse transcription–PCR. The templates were genomic DNA, RNA removed from genomic DNA, and cDNA, respectively. (**C**) Disruption of *msuB* affects the ability of *M. xanthus* to prey on *P. aeruginosa*. Predation assay on TPM (1.5% agar) plates. Scale bars = 10 mm. (**D**) The competition assay analyzed myxobacterial predation. After coculture of *M. xanthus* strains and PAO1 for 24 h, the cells were harvested and resuspended in 1 mL of MMC buffer. Subsequently, the cellular suspension was serially diluted in MMC buffer. For this, 3 µL of the suspensions (from 10^−1^ to 10^−6^) was spotted on LB agar plates supplemented with tetracycline (20 µg mL^−1^) from left to right. The plates were grown at 37 °C and photographed after 24 h. (**E**) Fruiting body formation was analyzed during the predation of *P. aeruginosa*. The plates were photographed by a stereomicroscope after 24 h of coculture. Scale bars = 500 µm.

Iron is essential for nearly all organisms and is also a crucial micronutrient that bacteria compete for [6]. Previous studies have demonstrated that siderophores can serve as competitive agents involved in microbial competition [6,43,44]. Here, we characterized a cluster of ferric siderophore uptake ABC transporter genes by performing BLAST searches in the *M. xanthus* DK1622 Genome Database. The cluster included four genes *MXAN_0684–MXAN_0687* (denoted *msuA–msuD*), which encoded an ATP-binding protein, two ABC transporter permease, and a ferric siderophore-binding protein, respectively (Figure 1A). Reverse transcription PCR analysis showed that the four genes were co-transcribed as an operon (Figure 1B). The myxobacterial predation ability and the effect of the *msuB* deletion were assessed in two ways: (i) by the lysed area of *P. aeruginosa* and (ii) by the ability to kill the *P. aeruginosa* prey. No significant difference was detected between the lysed area of *P. aeruginosa* caused by wild-type DK1622 and ∆*msuB/msuB* on the TPM (1.5% agar) plates, while the lysed area was significantly reduced in the ∆*msuB* mutant, suggesting that the deletion of *msuB* decreased the predation ability (Figure 1C). We further tested the ability to kill *P. aeruginosa* by a bacterial competition assay. As expected, the ∆*msuB* mutant showed a significantly reduced ability to kill the *P. aeruginosa* prey compared with wild-type DK1622 and ∆*msuB/msuB* (Figure 1D). We further found that the fruiting body development of the ∆*msuB* mutant was delayed in comparison with wild-type DK1622 and ∆*msuB/msuB* during predation on *P. aeruginosa* (Figure 1E). Interestingly, we observed a similar delay in the fruiting body development of the ∆*msuB* mutant on plain TPM (1.5% agar) plates (Figure S1), which depended on the programmed cell death of *M. xanthus* cells to provide nutrients to the fruiting/sporulating sub-population [45]. We could not rule out a general fitness disadvantage of the ∆*msuB* mutant on the TPM (1.5% agar) plates, and tested the effect of *musB* deletion on growth and gliding motility. The results showed that disruption of the *msuB* gene did not cause significant growth and motility defects in the presence of nutrients (0.5–1% CTT, 1.5% agar) (Figures S2 and S3). Taken together, these results suggest that MsuB affects predation in *M. xanthus*.

### 3.2. Deletion of msuB Leads to a Significant Decrease in Intracellular Iron Levels

**Figure 2:**
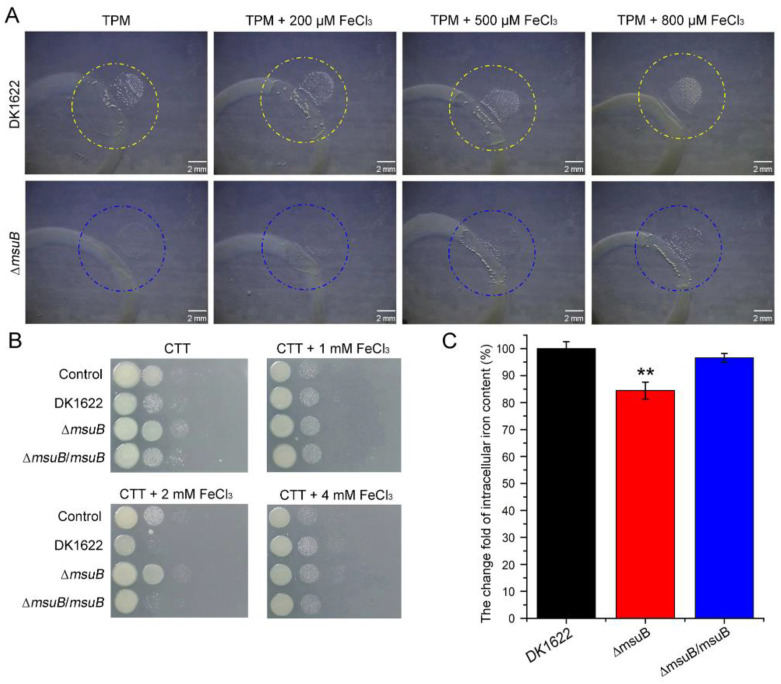
Differences in predation ability between DK1622 and ∆*msuB* mutants under different iron concentrations. (**A**) The effects of different iron concentrations on myxobacterial predation on TPM (1.5% agar) plates were assessed by a predation experiment. Scale bars = 2 mm. (**B**) The survival of *P. aeruginosa* co-cultured with DK1622, ∆*msuB*, and ∆*msuB/msuB* at different iron concentrations for 24 h was calculated by a competitive experiment. The cocultured *M. xanthus* strains and PAO1 were harvested and the cells were resuspended in 1 mL of MMC buffer. Subsequently, the cellular suspension was serially diluted in MMC buffer. Next, 3 µL of the suspensions (from 10^−2^ to 10^−6^) was spotted on LB agar plates supplemented with tetracycline (20 µg mL^−1^) from left to right. The plates were grown at 37 °C and photographed after 24 h. (**C**) The relative content of intracellular iron was detected by the BPS method. ** *p*-value < 0.01 compared with the DK1622 as measured by a two-tailed unpaired Student’s *t*-test.

Since the *msuB* gene encodes a permease of the ferric-siderophore uptake ABC transporter, it is reasonable that the deletion of *msuB* affects iron uptake. Thus, we hypothesized that intracellular iron levels may be essential for myxobacterial predation. To test this hypothesis, we performed a predation assay on TPM (1.5% agar) plates and a bacterial competition assay on CTT (1.5% agar) plates. The results suggested that the lysed area of *P. aeruginosa* gradually increased with an increase in the iron level in the medium in the ∆*msuB* mutant, and DK1622 showed the opposite trend on TPM (1.5% agar) plates (Figure 2A). Furthermore, we also found that the ability of DK1622 and ∆*msuB* mutants to kill *P. aeruginosa* increased with lower iron levels and decreased at a higher iron level on CTT (1.5% agar) plates. Especially, 1 mM FeCl_3_ increased the predation ability of ∆*msuB* mutants, and 2 mM FeCl_3_ significantly enhanced the ability of DK1622 to kill *P. aeruginosa* (Figure 2B). Intracellular iron levels were assessed by the bathophenanthroline disulfonate (BPS)-based colorimetric method. The results showed that disruption of the *msuB* gene resulted in a decrease in intracellular iron levels (Figure 2C). On the basis of these observations, we concluded that intracellular iron levels might be involved in regulating the predation of *M. xanthus*.

### 3.3. MsuB Is Involved in the Maintenance of Myxobacterial Intracellular Iron Homeostasis

**Figure 3:**
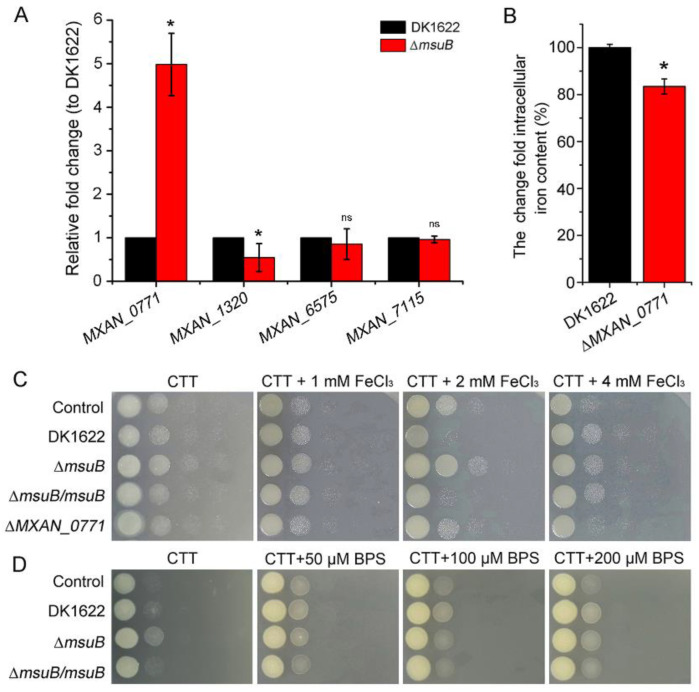
MsuB has a profound effect on iron homeostasis in *M. xanthus*. (**A**) qRT-PCR was used to measure the expression of other iron ABC transporter permease genes, which were normalized to the expression of *gapA*. DK1622 and the ∆*msuB* mutant were grown on CTT plates. * *p*-value < 0.01 compared with the DK1622; ns, not significant. (**B**) The intracellular iron level of ∆*MXAN_0771* mutant was detected by the BPS method. * *p*-value < 0.01 compared with the DK1622 as measured by a two-tailed unpaired Student’s *t*-test. (**C**) The competition assay analyzed the predation of different mutants on CTT (1.5% agar) plates containing different concentrations of FeCl_3_. After 24 h of coculture, the colony was harvested, and the cells were resuspended in 1 mL of MMC buffer. Subsequently, the cellular suspension was serially diluted in MMC buffer. Next, 3 µL of the suspension (from 10^−2^ to 10^−6^) was spotted on LB agar plates supplemented with tetracycline (20 µg mL^−1^) from left to right. The plates were grown at 37 °C and photographed after 24 h. (**D**) The competition assay analyzed the predation of mutants on CTT (1.5% agar) plates containing different concentrations of BPS. After 24 h of coculture, the colony was harvested, and the cells were resuspended in 1 mL of MMC buffer. Subsequently, the cellular suspension was serially diluted in MMC buffer, and then 3 µL of the suspension (from 10^−2^ to 10^−6^) was spotted on LB agar plates supplemented with tetracycline (20 µg mL^−1^) from left to right. The plates were grown at 37 °C and photographed after 24 h.

The results presented so far indicated that the Δ*msuB* mutant had a significantly reduced ability. In addition to the *msuABCD* gene cluster, there were four iron ABC transporters in *M. xanthus*’s genome. We further investigated the effect of deleting *msuB* on the expression levels of other iron ABC transporter permease genes. The results revealed that the expression of *MXAN_0771* was significantly upregulated in the ∆*msuB* mutant (Figure 3A). MXAN_0771 is predicted to be an ABC-type Fe^3+^ transport system, permease component [46]. We found that the intracellular iron level was also significantly decreased in the ∆*MXAN_0771* mutant, which was similar to the ∆*msuB* mutant (Figure 3B). However, the bacterial competition assay demonstrated that disruption of *MXAN_0771* did not result in a significant decrease in the ability to kill *P. aeruginosa*, even at CTT (1.5% agar) plates with different iron concentrations (Figure 3C). We also found that the deletion of *MXAN_0771* did not lead to a significant decrease in the lysed area of *P. aeruginosa* at TPM (1.5% agar) plates with different iron concentrations (Figure S4). Subsequently, we assessed the ability to kill *P. aeruginosa* under low iron levels and found that iron deficiency did not alter the ability of ∆*msuB* mutants to kill *P. aeruginosa*, even though iron deficiency led to a significant increase in the colony growth diameter of DK1622 (Figure 3D and Figure S5). These findings suggest that MsuB plays an important role in maintaining myxobacterial intracellular iron homeostasis, but iron deficiency might be not the major reason for the decreased predation ability of the ∆*msuB* mutant.

### 3.4. Disruption of Siderophore Synthesis and Transport Decreased Predation in M. xanthus

**Figure 4:**
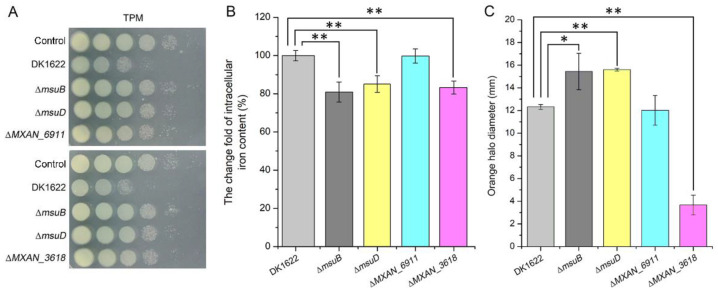
The effects of deleting siderophore synthesis and transport-related genes on intracellular iron levels, predation, and siderophore production. (**A**) The competition assay analyzed the predation of different mutants on TPM (1.5% agar) plates. After coculture of *M. xanthus* strains and PAO1 for 24 h, the cells were harvested and resuspended in 1 mL of MMC buffer. Subsequently, the cellular suspension was serially diluted in MMC buffer, and 3 µL of the suspension (from 10^−1^ to 10^−6^) was spotted on LB agar plates supplemented with tetracycline (20 µg mL^−1^) from left to right. The plates were grown at 37 °C and photographed after 24 h. (**B**) The relative content of intracellular iron was detected by the BPS method. ** *p*-value < 0.01 compared with DK1622 as measured by a two-tailed unpaired Student’s *t*-test. (**C**) Determination of siderophore production by the chromeazurol S overlay (O-CAS) assay. * *p*-value < 0.05 and ** *p*-value < 0.01 compared with DK1622 as measured by a two-tailed unpaired Student’s *t*-test.

To further elucidate the mechanistic details of the reduction in predation ability in the ∆*msuB* mutant, we constructed ∆*MXAN_3618*, ∆*MXAN_6911*, and ∆*msuD* mutants, which encoded non-ribosomal peptide synthetase, TonB-dependent receptor, and substrate-binding protein in charge of siderophore synthesis, recognition, and binding in *M. xanthus*, respectively. As expected, the disruption of *MXAN_3618* and *msuD* resulted in a significant decrease in the ability to kill *P. aeruginosa*, and the intracellular iron levels of ∆*MXAN_3618* and ∆*msuD* mutants also significantly decreased, consistent with the ∆*msuB* mutant (Figure 4A,B). Furthermore, the production of siderophores was detected by a chromeazurol S overlay (O-CAS) assay. The results showed that the deletion of *msuB* and *msuD* resulted in a significant increase in the diameter of the orange halo, while the deletion of *MXAN_3618* led to a decrease in the diameter of the orange halo compared with DK1622 (Figure 4C). Although deletion of *MXAN_6911* did not affect the intracellular iron levels and the production of siderophores, the ∆*MXAN_6911* mutant showed an attenuated ability to kill *P. aeruginosa* (Figure 4A–C). These results support the view that obstruction of the siderophore transport and synthesis pathway results in a reduction in predation in *M. xanthus*.

### 3.5. MsuABCD Is Closely Related to the Secretion of Lytic Enzymes

**Figure 5:**
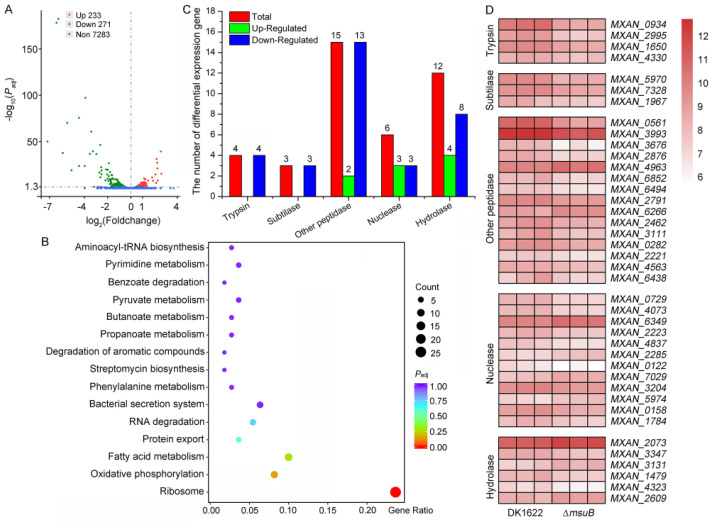
Global transcriptional profiles of DK1622 and the ∆*msuB* mutant. (**A**) Volcano plot of differentially expressed genes (DEGs). Red dots and green dots represent the up- and down-regulated genes with significant differences, respectively (*P*_adj_ < 0.05). The blue dots represent the genes that did not change significantly. (**B**) KEGG pathway enrichment results of DEGs. The *y*–axis represents the categories of the KEGG pathways. The *x*–axis is the richness factor (richness factor = amount of DEGs in the pathway divided by the amount of all genes in the background gene set). (**C**) Analysis of the DEGs encoding lytic enzymes. (**D**) Heatmap of RNA sequencing, showing the expression pattern of genes encoding lytic enzymes in DK1622 and the ∆*msuB* mutant, respectively. The normalized FPKM value of differentially expressed genes was used for plotting.

In order to shed light on the prevailing mechanistic uncertainties in siderophores’ interference with myxobacterial predation, we probed the overall transcriptional profile of DK1622 and ∆*msuB* mutants by transcriptome analysis. Our data showed that deletion of *msuB* significantly altered the gene expression profile, with 504 differentially expressed genes (DEGs), including 271 upregulated and 233 downregulated genes (Figure 5A, Supplementary Table S2). The upregulated genes were enriched in the ribosome, protein export, and bacterial secretion system pathways, and the deletion of *msuB* also enhanced the secretion of extracellular protein (Figure 5B and Figure S6). Surprisingly, most of these DEGs encoding lytic enzymes such as proteases, peptidases, nucleases, and hydrolase had significantly downregulated expression (Figure 5C). Among these, 13 DEGs encoding protease HtpX (MXAN_0561), M36 family metallopeptidase (MXAN_3676), S8 family serine peptidase (MXAN_5970), and trypsin-like peptidase (MXAN_2995) were downregulated by two- to fourfold and were involved in extracellular degradation of prey cells (Figure 5D). Collectively, these results suggest that the downregulation of lytic enzyme genes might impair the predation ability of the ∆*msuB* mutant.

### 3.6. Interruption of Siderophore Transport Decreased the Expression of OMV-Specific Proteins

**Figure 6:**
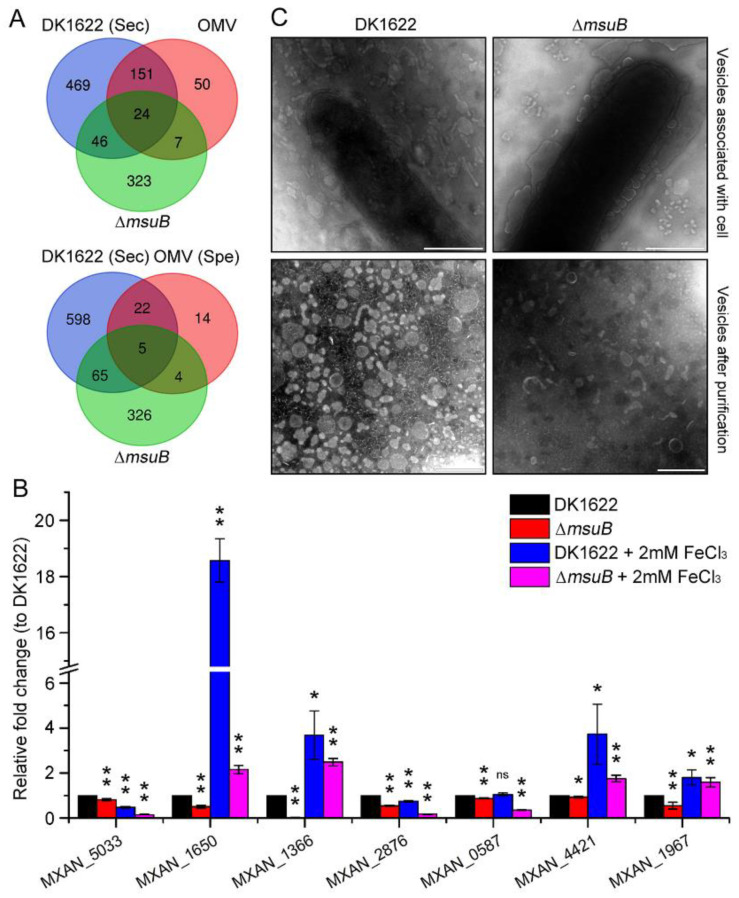
Effects of *msuB* deletion on the expression of OMV-specific proteins in *M. xanthus*. (**A**) Venn diagram analysis of DEGs and the genes encoding secretion proteins (DK1622) and OMV proteins. (**B**) DK1622 and ∆*msuB* mutants were grown in CTT medium or CTT medium with 2 mM FeCl_3_ for 24 h at 30 °C. The sample was harvested by centrifuging 12,000× *g* at 4 °C and total RNA was extracted for detecting the expression of OMV related-genes, which were normalized to the expression of *gapA*. * *p*-value < 0.05; ** *p*-value < 0.01 compared with DK1622; ns, not significant. (**C**) DK1622 and the ∆*msuB* mutant were grown in a CTT medium for 24 h at 30 °C. OMVs were observed by transmission electron microscopy (TEM) (scale bar = 500 nm) (top panel). OMVs were isolated by centrifuging at 150,000× *g* and were observed by transmission electron microscopy (TEM) (scale bar = 200 nm) (bottom panel).

Several studies have suggested that outer membrane vesicles (OMVs) play an important role in the delivery of lytic factors onto the prey cells [15,31,47]. On the basis of the DK1622 secretion proteins (Supplementary Table S3) and public proteomic data sets [31], the common DEGs between secretion protein and OMV protein were analyzed. The results showed that nine DEGs were common, including that five OMV-specific proteins, which encode two trypsin-like serine proteases, two S8 family serine peptidases, and one endopeptidase (Figure 6A and Figure S7, Table 1). Our results confirmed that the expression of genes related to OMVs’ lytic factors was significantly decreased in the ∆*msuB* mutant (Figure 6B). Some of the DEGs, which encoded trypsin-like serine protease (MXAN_1650) and S8 family serine peptidase (MXAN_1967), were significantly upregulated under iron-rich conditions in DK1622, implying that iron increased myxobacterial predation by activating the OMVs’ lytic factors. To investigate the effect of deleting *msuB* on the formation of OMVs, the morphology of the OMVs was observed by transmission electron microscopy (TEM). The results found that the deletion of *msuB* reduced OMVs’ biogenesis (Figure 6C). Together, these results demonstrate that the disruption of siderophore transport results in the reduction in the predation ability of *M. xanthus* by affecting the expression of OMV-specific proteins.

## 4. Discussion

Scarce nutrients have been confirmed to be crucial driving forces that shape the composition of the microbial community [3,48,49]. Iron is a scarce essential nutrient that bacteria compete for in many niches. *M. xanthus* can generate specialized cells by phase variation under iron limitations [50]. These specialized cells decrease the biosynthesis of antibiotics and pigments and increase expression of the siderophores, hemin binding proteins, and iron transport proteins for acquiring iron. Our results showed that the color of colonies was different between DK1622 and ∆*msuB* mutants, and the deletion of the *msuB* gene led to a decrease in intracellular iron level and an increase in the production of siderophores (Figure S2 and Figure 4C). Most bacteria use siderophores to chelate ferric iron (Fe^3+^), and iron–siderophore complexes are transported via the metal-type ABC transporter [51,52]. There are 57 complete ABC transporters in the genome of *M. xanthus*, including 20 importers and 37 exporters [53]. On the basis of our bioinformatic analysis, we found that five ABC transporters might be involved in iron uptake in *M. xanthus*. In this study, we investigated the functions of two ABC transporters in myxobacterial predation by constructing in-frame deletion mutants, MsuABCD and MXAN_0770–MXAN_0772. The results showed that deletion of the *msuB* and *MXAN_0771* genes caused a dramatic decrease in intracellular iron levels compared with DK1622 (Figure 2B and Figure 3B). However, there was an obvious difference between the ∆*msuB* and ∆*MXAN_0771* mutants in their predation ability (Figure 3C). A previous study demonstrated that virus capsid-like nano-compartments are assembled for storing iron and protecting cells from oxidative stress in *M. xanthus* [54], implying that fluctuations in intracellular iron are not the major determinants of reduced myxobacterial predation. Actually, deletion of the *msuB* gene significantly altered the gene expression profile (Figure 5A). Therefore, we hypothesized that it may be attributed to siderophores transport. According to our analysis of the mutant (∆*MXAN_3618*), we found that disruption of siderophore biosynthesis led to a reduction in predation ability (Figure 4B). According to an analysis of the siderophore content secreted by the ∆*msuB*, ∆*msuD*, and ∆*MXAN_3618* mutants, it was further confirmed that the interruption of siderophore transport resulted in a decrease in predation ability in *M. xanthus*.

As mentioned above, siderophores are involved in myxobacterial predation. However, this also raises an important question of how siderophore transport alters myxobacterial predation behavior. To address this question, we used transcriptome analysis to show that the upregulated DEGs were significantly enriched in the ribosome, protein export, and bacterial secretion systems (Figure 5B), and extracellular proteins obviously increased in the ∆*msuB* mutant (Figure S6). These results implied that loss of *msuB* may affect the synthesis and export of protein. In bacteria, the Sec machinery is responsible for translocating proteins across the cytoplasmic membrane [55]. Our data showed that *MXAN_4691*, *MXAN_4692*, and *MXAN_7509* were distinctively upregulated, which encode SecD, YajC, and YidC, respectively. The three proteins form a complex with SecYEG in vivo known as the holotranslocon and assist the core Sec machinery [56]. The unfolded polypeptides are mainly secreted through the Sec translocon, and the fully folded proteins are transported through the twin-arginine translocation translocase [57,58]. A previous study found that Fur regulon was involved in secretory pathways by binding to the promoter of *secY* in *Neisseria gonorrhoeae* [59]. Even though the ferric uptake regulator (Fur) regulon has not been identified in *M. xanthus*, we suggested that deletion of the *msuB* gene might alter the expression of Fur, thereby indirectly affecting the Sec-dependent secretion of extracellular proteins (Figure S6). *M. xanthus* cells form thin biofilms which facilitate predation and food gathering. The cluster of cells cooperates to produce a large number of digestive enzymes, which digest prokaryotic and eukaryotic microorganisms [17,60]. Surprisingly, some lytic enzymes were significantly downregulated in the ∆*msuB* mutant, including proteases (MXAN_1650), peptidases (MXAN_7328, MXAN_1967), nucleases (MXAN_3347, MXAN_4323), and hydrolase (MXAN_4073, MXAN_4837) (Figure 5C,D). Prey cells can be lysed by using antibiotics, hydrolytic enzymes, and extracellular OMVs that may facilitate delivery [17]. MXAN_3564 (mepA), an M36 protease homolog, contributes to predation by degrading the proteins that are released from prey cells [31]. The enzymes with peptidoglycan-degrading activity have emerged as a class of antimicrobial proteins against pathogens by inducing prey cell lysis [61]. β-1,6-Glucanase GluM from the *Corallococcus* sp. strain EGB was essential for lysing the chitinous cell wall of certain fungi [62]. *M. xanthus* secretes a glycoside hydrolase 19 family LlpM with lysozyme-like activity, which displays bacteriolytic activity in vivo and in vitro [47]. Thus, our results support the conclusion that a reduction in the secretion of lytic enzymes leads to a decrease in predation ability in ∆*msuB* mutants.

OMVs are involved in multiple biological processes, including bacterial competition and nutrition acquisition, through delivering various biologically active molecules in high concentrations [63,64]. OMVs are a unique bacterial secretion pathway, termed Type 0 secretion system (T0SS) [65]. Transcriptome analysis showed that the OMVs secreted by *M. xanthus* could induce changes in the expression of large numbers of *E. coli* genes [66]. In *P. aeruginosa*, the Type VI secretion system effector TseF secreted by H3-T6SS can be incorporated into OMVs. TseF can be recognized by the TonB-dependent iron transporter FptA, and then facilitates the transport of iron into the cell via the OMV complex [67,68]. TonB-dependent receptors, such as CirA and MXAN_6911, have also been found in *E. coli* and *M. xanthus* OMVs, respectively [31,69]. Our results suggested that deletion of the *MXAN_6911* gene led to a decrease in predation ability, although it had no effect on intracellular iron levels and siderophore synthesis (Figure 4A–C). These results further confirm that the disruption of siderophore transport results in a decrease in predation ability in *M. xanthus*. By using reversed phase liquid chromatography mass spectrometry (RP LC/MS) analysis, Berleman et al. identified a conservative set of 46 OMV-specific and 188 OMV-contained proteins from the purifying vesicles and whole cell membranes [31]. The siderophores myxochelin A and myxochelin B were also identified in OMVs by LC/MS [31]. On basis of these data, we explored the relationship between OMV-related proteins and proteins encoded by DEGs. The results demonstrated that 31 common DEGs were identified, including 9 DEGs encoding OMV-specific proteins (Figure 6A). Interestingly, the expression of nine DEGs was significantly downregulated, implying that the interruption of siderophore transport affected the production of OMV-specific proteins. Taken together, these results demonstrated that siderophores manipulate myxobacterial predation by interfering with the expression of outer membrane vesicle proteins.

## Figures and Tables

**Table 1:** OMV-specific expression genes.

No.	Old Locus	Locus	Gene Function
1	*MXAN_0587*	*MXAN_RS02845*	trypsin-like serine protease: Trypsin
2	*MXAN_1967*	*MXAN_RS09535*	S8 family serine peptidase: Subtilase family
3	*MXAN_1650*	*MXAN_RS08015*	trypsin-like serine protease: Trypsin
4	*MXAN_2876*	*MXAN_RS13930*	endopeptidase
5	*MXAN_5970*	*MXAN_RS28960*	S8 family serine peptidase: Subtilase family

## Data Availability

The original contributions presented in the study are included in the article/Supplementary Materials, further inquiries can be directed to the corresponding authors. The raw data associated with this study has been deposited in the Sequence Read Archive (SRA) under the accession PRJNA849267.

## Supplementary Materials

The following supporting information can be downloaded at: https://www.mdpi.com/2073-4409/11/23/3718/s1, Figure S1: Effect of *msuB* deletion on *M. xanthus* fruiting body development. Figure S2: Effect of *msuB* deletion on *M. xanthus* growth. Colony diameter (A) and morphology (B) of DK1622 and ∆*msuB* mutants was measured on CTT (1.5% agar) plates. Figure S3: Effect of *msuB* deletion on *M. xanthus* gliding motility and exopolysaccharide accumulation. (A) Strains were incubated on 0.5% CTT with 1.5% agar to detect gliding motility. Scale bars, 5 mm (top plane), 1 mm (down plane). The same results were obtained in two independent experiments. (B) Lack of MsuB does not affect exopolysaccharide accumulation. Δ*pilA* strain served as negative control. The same results were obtained in two independent experiments. Figure S4: The effects of different iron concentrations on myxobacterial predation on TPM (1.5% agar) plates were assessed by a predation experiment. Scale bars = 2 mm. Figure S5: Effect of *msuB* deletion on *M. xanthus* growth under low iron level. Colony diameter of DK1622 and ∆*msuB* mutants was measured on CTT (1.5% agar) plates with different concentration of BPS (bathophenanthroline disulfonate). Figure S6: Effect of *msuB* deletion on extracellular proteins. DK1622 and ∆*msuB* mutants were grown in CTT medium for 24 h at 30 °C, 200 rpm. The optical density (OD_550nm_) of DK1622 and ∆*msuB* mutants reached to 0.8. The supernatants were obtained by centrifuging at 12,000× *g*. The extracellular proteins were precipitated with ammonium sulphate and resuspended in PBS. The samples were detected by SDS-PAGE. Loading volumes were 10 µL (A), 20 µL (B), and 30 µL (C), respectively. Figure S7: The expression of OMVs related genes in ∆*msuB* mutant, compared with DK1622. * *p*-value < 0.05 and ** *p*-value < 0.01 compared with the DK1622 as measured by a two-tailed unpaired Student’s *t*-test. Table S1: Strains and plasmids used in this work; Table S2: Deletion of msuB significantly altered the differentially expressed genes (DEGs); Table S3: The secretion proteins derived from wild type DK1622.

## References

[1] Klein T.A., Ahmad S., Whitney J.C. (2020). Contact-dependent interbacterial antagonism mediated by protein secretion machines. Trends Microbiol..

[2] Stubbendieck R.M., Straight P.D. (2016). Multifaceted interfaces of bacterial competition. J. Bacteriol..

[3] Hibbing M.E., Fuqua C., Parsek M.R., Peterson S.B. (2010). Bacterial competition: Surviving and thriving in the microbial jungle. Nat. Rev. Microbiol..

[4] Ghoul M., Mitri S. (2016). The ecology and evolution of microbial competition. Trends Microbiol..

[5] Kamal F., Liang X., Manera K., Pei T.T., Kim H., Lam L.G., Pun A., Hersch S.J., Dong T.G. (2020). Differential cellular response to translocated toxic effectors and physical penetration by the Type VI Secretion System. Cell Rep..

[6] Kramer J., Ozkaya O., Kummerli R. (2020). Bacterial siderophores in community and host interactions. Nat. Rev. Microbiol..

[7] Kurth C., Kage H., Nett M. (2016). Siderophores as molecular tools in medical and environmental applications. Org. Biomol. Chem..

[8] Gorska A., Sloderbach A., Marszall M.P. (2014). Siderophore-drug complexes: Potential medicinal applications of the ‘Trojan horse’ strategy. Trends Pharmacol. Sci..

[9] Clarke T.E., Tari L.W., Vogel H.J. (2001). Structural biology of bacterial iron uptake systems. Curr. Top. Med. Chem..

[10] Eickhoff M.J., Bassler B.L. (2020). *Vibrio fischeri* siderophore production drives competitive exclusion during dual-species growth. Mol. Microbiol..

[11] Leinweber A., Weigert M., Kummerli R. (2018). The bacterium *Pseudomonas aeruginosa* senses and gradually responds to interspecific competition for iron. Evolution.

[12] Sutton D., Livingstone P.G., Furness E., Swain M.T., Whitworth D.E. (2019). Genome-wide identification of myxobacterial predation genes and demonstration of formaldehyde secretion as a potentially predation-resistant trait of *Pseudomonas aeruginosa*. Front. Microbiol..

[13] Akbar S., Phillips K.E., Misra S.K., Sharp J.S., Stevens D.C. (2022). Differential response to prey quorum signals indicates predatory specialization of myxobacteria and ability to predate *Pseudomonas aeruginosa*. Environ. Microbiol..

[14] Livingstone P.G., Morphew R.M., Whitworth D.E. (2017). Myxobacteria are able to prey broadly upon clinically-relevant pathogens, exhibiting a prey range which cannot be explained by phylogeny. Front. Microbiol..

[15] Thiery S., Kaimer C. (2020). The predation strategy of *Myxococcus xanthus*. Front. Microbiol..

[16] Morgan A.D., MacLean R.C., Hillesland K.L., Velicer G.J. (2010). Comparative analysis of *Myxococcus* predation on soil bacteria. Appl. Environ. Microbiol..

[17] Keane R., Berleman J. (2016). The predatory life cycle of *Myxococcus xanthus*. Microbiology.

[18] Velicer G.J., Kroos L., Lenski R.E. (2000). Developmental cheating in the social bacterium *Myxococcus xanthus*. Nature.

[19] Stolle A.S., Meader B.T., Toska J., Mekalanos J.J. (2021). Endogenous membrane stress induces T6SS activity in *Pseudomonas aeruginosa*. Proc. Natl. Acad. Sci. USA.

[20] Kunze B., Bedorf N., Kohl W., Hofle G., Reichenbach H. (1989). Myxochelin A, a new iron-chelating compound from *Angiococcus disciformis* (Myxobacterales). Production, isolation, physico-chemical and biological properties. J. Antibiot..

[21] Silakowski B., Kunze B., Nordsiek G., Blocker H., Hofle G., Muller R. (2000). The myxochelin iron transport regulon of the myxobacterium *Stigmatella aurantiaca* Sg a15. Eur. J. Biochem..

[22] Gaitatzis N., Kunze B., Muller R. (2005). Novel insights into siderophore formation in myxobacteria. Chembiochem.

[23] Findlay B.L. (2016). The chemical ecology of predatory soil bacteria. ACS Chem. Biol..

[24] Krug D., Zurek G., Revermann O., Vos M., Velicer G.J., Muller R. (2008). Discovering the hidden secondary metabolome of *Myxococcus xanthus*: A study of intraspecific diversity. Appl. Environ. Microbiol..

[25] Weissman K.J., Muller R. (2010). Myxobacterial secondary metabolites: Bioactivities and modes-of-action. Nat. Prod. Rep..

[26] Sester A., Winand L., Pace S., Hiller W., Werz O., Nett M. (2019). Myxochelin- and Pseudochelin-derived lipoxygenase inhibitors from a genetically engineered *Myxococcus xanthus* strain. J. Nat. Prod..

[27] Lee N., Kim W., Chung J., Lee Y., Cho S., Jang K.S., Kim S.C., Palsson B., Cho B.K. (2020). Iron competition triggers antibiotic biosynthesis in *Streptomyces coelicolor* during coculture with *Myxococcus xanthus*. ISME J..

[28] Dhurve G., Madikonda A.K., Jagannadham M.V., Siddavattam D. (2022). Outer membrane vesicles of *Acinetobacter baumannii* DS002 are selectively enriched with TonB-Dependent transporters and play a key role in iron acquisition. Microbiol. Spectr..

[29] Li C., Zhu L., Wang D., Wei Z., Hao X., Wang Z., Li T., Zhang L., Lu Z., Long M. (2022). T6SS secretes an LPS-binding effector to recruit OMVs for exploitative competition and horizontal gene transfer. ISME J..

[30] Evans A.G.L., Davey H.M., Cookson A., Currinn H., Cooke-Fox G., Stanczyk P.J., Whitworth D.E. (2012). Predatory activity of *Myxococcus xanthus* outer-membrane vesicles and properties of their hydrolase cargo. Microbiology.

[31] Berleman J.E., Allen S., Danielewicz M.A., Remis J.P., Gorur A., Cunha J., Hadi M.Z., Zusman D.R., Northen T.R., Witkowska H.E. (2014). The lethal cargo of *Myxococcus xanthus* outer membrane vesicles. Front. Microbiol..

[32] Kaiser D. (1979). Social gliding is correlated with the presence of pili in *Myxococcus xanthus*. Proc. Natl. Acad. Sci. USA.

[33] Yang Y.J., Wang Y., Li Z.F., Gong Y., Zhang P., Hu W.C., Sheng D.H., Li Y.Z. (2017). Increasing on-target cleavage efficiency for CRISPR/Cas9-induced large fragment deletion in *Myxococcus xanthus*. Microb. Cell Fact..

[34] Julien B., Kaiser A.D., Garza A. (2000). Spatial control of cell differentiation in *Myxococcus xanthus*. Proc. Natl. Acad. Sci. USA.

[35] Wu S.S., Wu J., Kaiser D. (1997). The *Myxococcus xanthus* pilT locus is required for social gliding motility although pili are still produced. Mol. Microbiol..

[36] Dong H., Xu X., Gao R., Li Y., Li A., Yao Q., Zhu H. (2021). *Myxococcus xanthus* R31 suppresses tomato bacterial wilt by inhibiting the pathogen *Ralstonia solanacearum* with secreted proteins. Front. Microbiol..

[37] Berleman J.E., Chumley T., Cheung P., Kirby J.R. (2006). Rippling is a predatory behavior in *Myxococcus xanthus*. J. Bacteriol..

[38] Basler M., Ho B.T., Mekalanos J.J. (2013). Tit-for-tat: Type VI secretion system counterattack during bacterial cell-cell interactions. Cell.

[39] Hsu P.C., Yang C.Y., Lan C.Y. (2011). *Candida albicans* Hap43 is a repressor induced under low-iron conditions and is essential for iron-responsive transcriptional regulation and virulence. Eukaryot. Cell.

[40] Dong Y., Zhang D., Yu Q., Zhao Q., Xiao C., Zhang K., Jia C., Chen S., Zhang B., Zhang B. (2017). Loss of Ssq1 leads to mitochondrial dysfunction, activation of autophagy and cell cycle arrest due to iron overload triggered by mitochondrial iron-sulfur cluster assembly defects in *Candida albicans*. Int. J. Biochem. Cell Biol..

[41] Kominek J., Doering D.T., Opulente D.A., Shen X.X., Zhou X., DeVirgilio J., Hulfachor A.B., Groenewald M., McGee M.A., Karlen S.D. (2019). Eukaryotic acquisition of a bacterial operon. Cell.

[42] Livak K.J., Schmittgen T.D. (2001). Analysis of relative gene expression data using real-time quantitative PCR and the 2^−ΔΔCT^ Method. Methods.

[43] Niehus R., Picot A., Oliveira N.M., Mitri S., Foster K.R. (2017). The evolution of siderophore production as a competitive trait. Evolution.

[44] Lee W., van Baalen M., Jansen V.A. (2016). Siderophore production and the evolution of investment in a public good: An adaptive dynamics approach to kin selection. J. Theor. Biol..

[45] Popp P.F., Mascher T. (2019). Coordinated cell death in isogenic bacterial populations: Sacrificing some for the benefit of many?. J. Mol. Biol..

[46] Goldman B.S., Nierman W.C., Kaiser D., Slater S.C., Durkin A.S., Eisen J.A., Ronning C.M., Barbazuk W.B., Blanchard M., Field C. (2006). Evolution of sensory complexity recorded in a myxobacterial genome. Proc. Natl. Acad. Sci. USA.

[47] Arend K.I., Schmidt J.J., Bentler T., Luchtefeld C., Eggerichs D., Hexamer H.M., Kaimer C. (2020). *Myxococcus xanthus* predation of Gram-positive or Gram-negative bacteria is mediated by different bacteriolytic mechanisms. Appl. Environ. Microbiol..

[48] Kamada N., Chen G.Y., Inohara N., Nunez G. (2013). Control of pathogens and pathobionts by the gut microbiota. Nat. Immunol..

[49] Knauf G.A., Powers M.J., Herrera C.M., Trent M.S., Davies B.W. (2022). Acinetobactin-mediated inhibition of commensal bacteria by *Acinetobacter baumannii*. mSphere.

[50] Dziewanowska K., Settles M., Hunter S., Linquist I., Schilkey F., Hartzell P.L. (2014). Phase variation in *Myxococcus xanthus* yields cells specialized for iron sequestration. PLoS ONE.

[51] Huang W., Wilks A. (2017). Extracellular heme uptake and the challenge of bacterial cell membranes. Annu. Rev. Biochem..

[52] Andrews S.C., Robinson A.K., Rodriguez-Quinones F. (2003). Bacterial iron homeostasis. FEMS Microbiol. Rev.

[53] Yan J., Bradley M.D., Friedman J., Welch R.D. (2014). Phenotypic profiling of ABC transporter coding genes in *Myxococcus xanthus*. Front. Microbiol..

[54] McHugh C.A., Fontana J., Nemecek D., Cheng N., Aksyuk A.A., Heymann J.B., Winkler D.C., Lam A.S., Wall J.S., Steven A.C. (2014). A virus capsid-like nanocompartment that stores iron and protects bacteria from oxidative stress. EMBO J..

[55] Dalal K., Duong F. (2011). The SecY complex: Conducting the orchestra of protein translocation. Trends Cell Biol..

[56] Cranford-Smith T., Huber D. (2018). The way is the goal: How SecA transports proteins across the cytoplasmic membrane in bacteria. FEMS Microbiol. Lett..

[57] Costa T.R., Felisberto-Rodrigues C., Meir A., Prevost M.S., Redzej A., Trokter M., Waksman G. (2015). Secretion systems in Gram-negative bacteria: Structural and mechanistic insights. Nat. Rev. Microbiol..

[58] Goosens V.J., van Dijl J.M. (2017). Twin-Arginine protein translocation. Curr. Top. Microbiol. Immunol..

[59] Sebastian S., Agarwal S., Murphy J.R., Genco C.A. (2002). The gonococcal fur regulon: Identification of additional genes involved in major catabolic, recombination, and secretory pathways. J. Bacteriol..

[60] Zusman D.R., Scott A.E., Yang Z., Kirby J.R. (2007). Chemosensory pathways, motility and development in *Myxococcus xanthus*. Nat. Rev. Microbiol..

[61] Sobieraj A.M., Huemer M., Zinsli L.V., Meile S., Keller A.P., Rohrig C., Eichenseher F., Shen Y., Zinkernagel A.S., Loessner M.J. (2020). Engineering of long-circulating peptidoglycan hydrolases enables efficient treatment of systemic *Staphylococcus aureus* infection. mBio.

[62] Li Z., Ye X., Liu M., Xia C., Zhang L., Luo X., Wang T., Chen Y., Zhao Y., Qiao Y. (2019). A novel outer membrane β-1,6-glucanase is deployed in the predation of fungi by myxobacteria. ISME J..

[63] Biller S.J., Schubotz F., Roggensack S.E., Thompson A.W., Summons R.E., Chisholm S.W. (2014). Bacterial vesicles in marine ecosystems. Science.

[64] Rivera J., Cordero R.J., Nakouzi A.S., Frases S., Nicola A., Casadevall A. (2010). *Bacillus anthracis* produces membrane-derived vesicles containing biologically active toxins. Proc. Natl. Acad. Sci. USA.

[65] Guerrero-Mandujano A., Hernandez-Cortez C., Ibarra J.A., Castro-Escarpulli G. (2017). The outer membrane vesicles: Secretion system type zero. Traffic.

[66] Livingstone P.G., Millard A.D., Swain M.T., Whitworth D.E. (2018). Transcriptional changes when *Myxococcus xanthus* preys on *Escherichia coli* suggest myxobacterial predators are constitutively toxic but regulate their feeding. Microb. Genom..

[67] Lin J., Zhang W., Cheng J., Yang X., Zhu K., Wang Y., Wei G., Qian P.Y., Luo Z.Q., Shen X. (2017). A *Pseudomonas* T6SS effector recruits PQS-containing outer membrane vesicles for iron acquisition. Nat. Commun..

[68] Behrens H.M., Lowe E.D., Gault J., Housden N.G., Kaminska R., Weber T.M., Thompson C.M.A., Mislin G.L.A., Schalk I.J., Walker D. (2020). Pyocin S5 import into *Pseudomonas aeruginosa* reveals a generic mode of bacteriocin transport. mBio.

[69] Lee E.Y., Bang J.Y., Park G.W., Choi D.S., Kang J.S., Kim H.J., Park K.S., Lee J.O., Kim Y.K., Kwon K.H. (2007). Global proteomic profiling of native outer membrane vesicles derived from *Escherichia coli*. Proteomics.

