# Supplementary material for: A Disturbed Siderophore Transport Inhibits Myxobacterial Predation
Source: Cells. 2022 Nov 22;11(23):3718. doi: 10.3390/cells11233718 (PMC9738627; doi:10.3390/cells11233718)
Supplement: Supplementary file 1 [file cells-11-03718-s001.zip › cells-1942367-supplementary/Supplementary Figures.pdf]

Supplementary Figures and legends

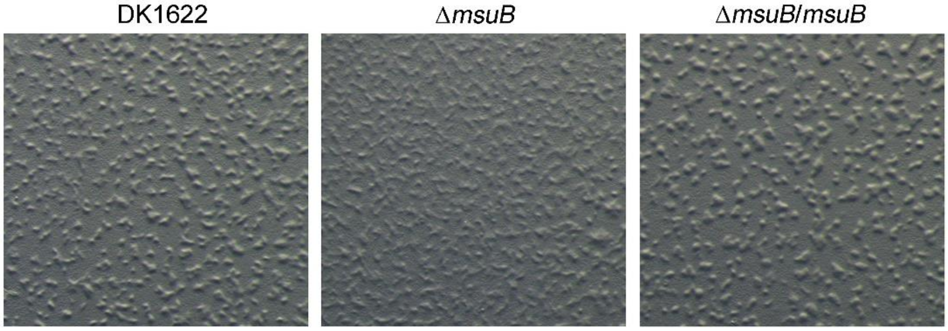

Figure S1. Effect of *msuB* deletion on *M. xanthus* fruiting body development.

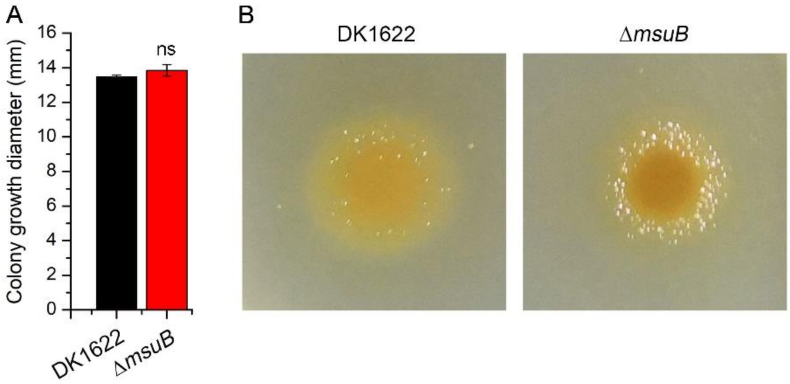

Figure S2. Effect of *msuB* deletion on *M. xanthus* growth. Colony diameter (A) and morphology (B) of DK1622 and Δ*msuB* mutants was measured on CTT (1.5% agar) plates.

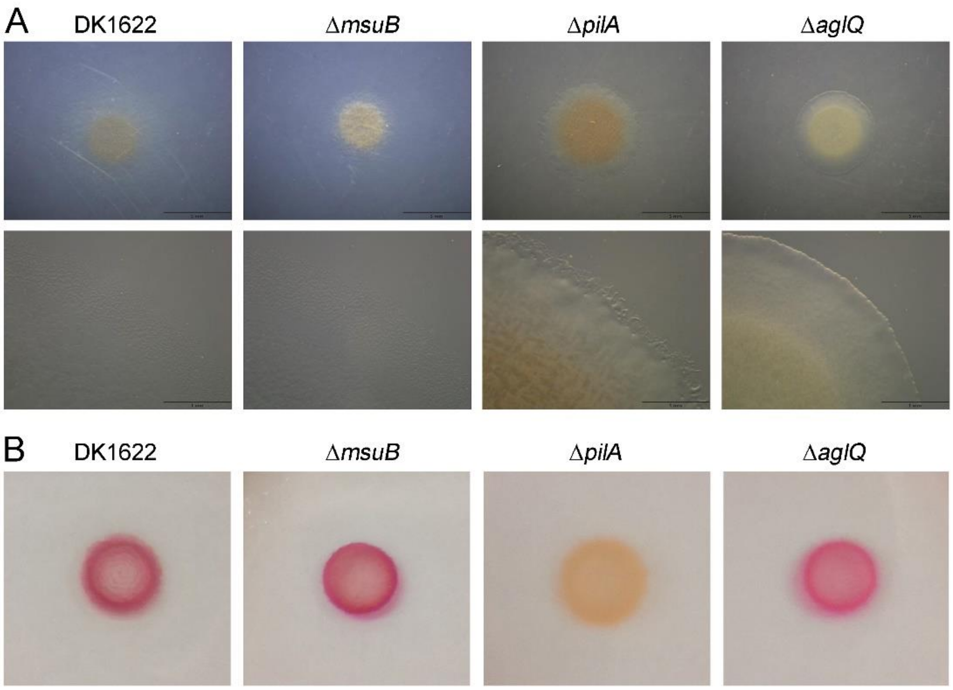

Figure S3. Effect of *msuB* deletion on *M. xanthus* gliding motility and exopolysaccharide accumulation. (A) Strains were incubated on 0.5% CTT with 1.5% agar to detect gliding motility. Scale bars, 5 mm (top plane), 1 mm (down plane). The same results were obtained in two independent experiments. (B) Lack of MsuB does not affect exopolysaccharide accumulation. Δ*pilA* strain served as negative control. The same results were obtained in two independent experiments.

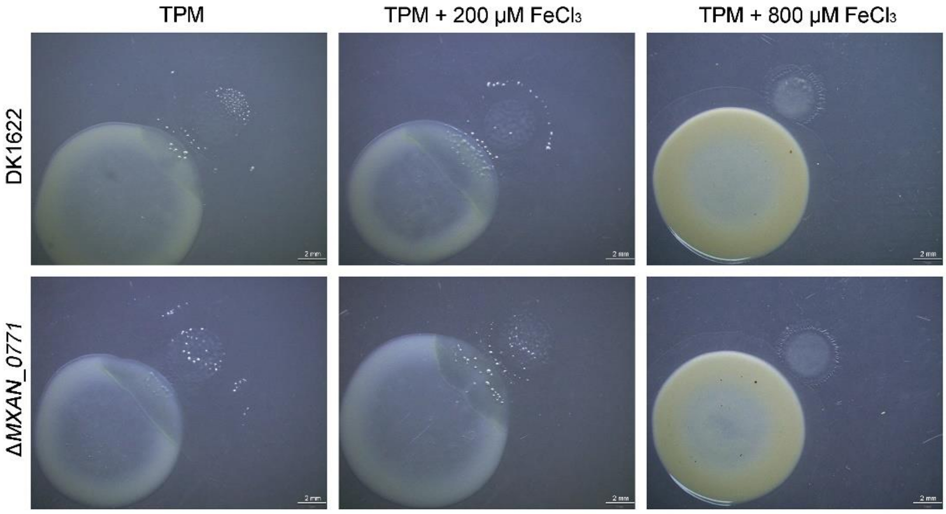

Figure S4. The effects of different iron concentrations on myxobacterial predation on TPM (1.5% agar) plates were assessed by a predation experiment. Scale bars = 2 mm.

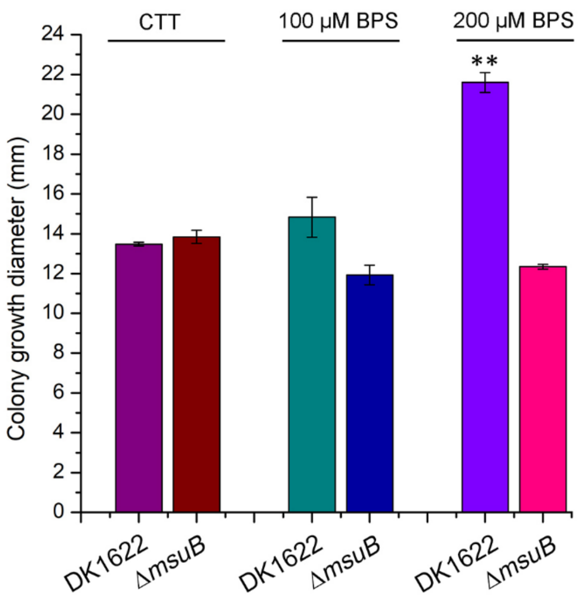

Figure S5. Effect of *msuB* deletion on *M. xanthus* growth under low iron level. Colony diameter of DK1622 and Δ*msuB* mutants was measured on CTT (1.5% agar) plates with different concentrations of BPS (bathophenanthroline disulfonate).

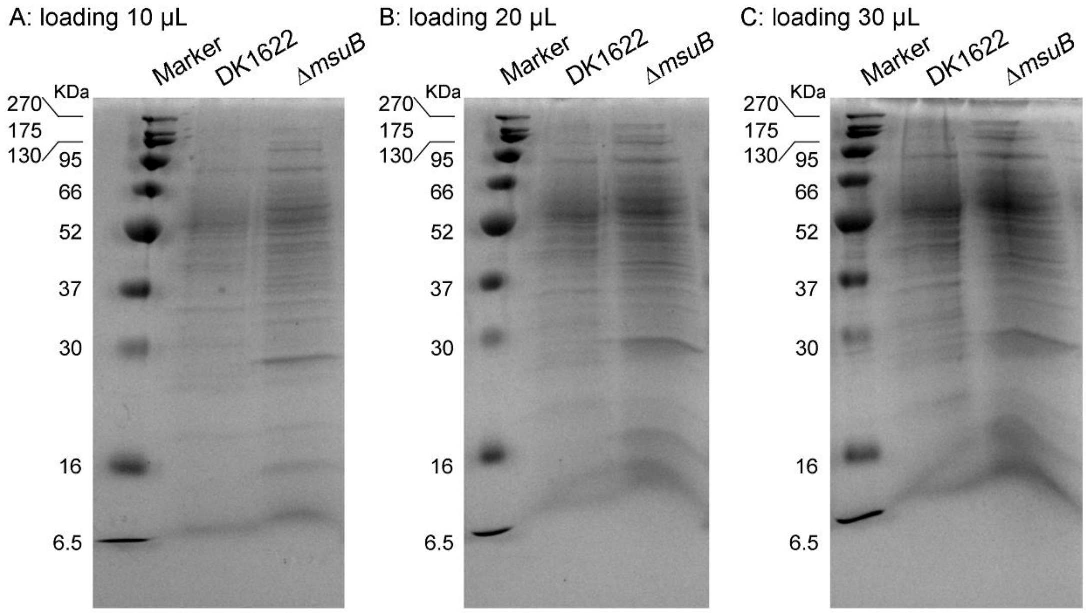

Figure S6. Effect of *msuB* deletion on extracellular proteins. DK1622 and Δ*msuB* mutants were grown in CTT medium for 24 h at 30 °C, 200 rpm. The optical density (OD_{550nm}) of DK1622 and Δ*msuB* mutants reached to 0.8. The supernatants were obtained by centrifuging at 12000×g. The extracellular proteins were precipitated with ammonium sulphate and resuspended in PBS. The samples were detected by SDS-PAGE. Loading volumes were 10 μL (A), 20 μL (B), and 30 μL (C), respectively.

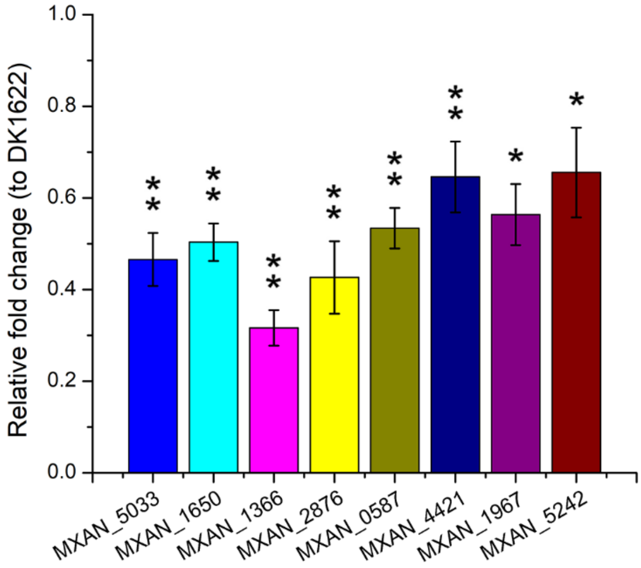

Figure S7. The expression of OMVs related genes in Δ*msuB* mutant, compared with DK1622. **P-value* <0.05 and ***P-value* <0.01 compared with the DK1622 as measured by a two-tailed unpaired Student's t-test.
