# Supplementary material for: A Disturbed Siderophore Transport Inhibits Myxobacterial Predation
Source: Cells. 2022 Nov 22;11(23):3718. doi: 10.3390/cells11233718 (PMC9738627; doi:10.3390/cells11233718)
Supplement: Supplementary file 1 [file cells-11-03718-s001.zip › cells-1942367-supplementary/Supplementary Table S1.pdf]

Supplementary Table S1. Strains and plasmids used in this work.

**Strains**		
**Strain**	**Genotype**	**Reference**
DK1622	Wildtype	[1]
Δ*msuB*	DK1622 Δ*msuB*	This study
Δ*msuB*/*msuB*	Δ*msuB* with *msuB* (wild type), Tet^r	This study
Δ*msuD*	DK1622 Δ*msuD*	This study
Δ*msuD*/*msuD*	Δ*msuD* with *msuD* (wild type), Tet^r	This study
Δ*MXAN_0771*	DK1622 Δ*MXAN_0771*	This study
Δ*MXAN_3618*	DK1622 Δ*MXAN_3618*	This study
Δ*MXAN_6911*	DK1622 Δ*MXAN_6911*	This study
**Plasmids**		
**Plasmid**	**Description**	**Reference**
pBJ113	Km^r, *galK*	[2,3]
pSWU30	Site specific integration vector with Mx8 attB, Tet^r	[4]
pBJ113-msuB	pBJ113, in-frame deletion construct for *msuB*, Km^r	This study
pBJ113-msuD	pBJ113, in-frame deletion construct for *msuD*, Km^r	This study
pBJ113-MXAN_0771	pBJ113, in-frame deletion construct for *MXAN_0771*, Km^r	This study
pBJ113-MXAN_3618	pBJ113, in-frame deletion construct for *MXAN_3618*, Km^r	This study
pBJ113-MXAN_6911	pBJ113, in-frame deletion construct for *MXAN_6911*, Km^r	This study
pSWU30-msuB	pSWU30, P_{nat} *msuB*, Site specific integration vector with Mx8 attB, Tet^r	This study
pSWU30-msuD	pSWU30, P_{nat} *msuD*, Site specific integration vector with Mx8 attB, Tet^r	This study

Reference

1. Kaiser, D. Social gliding is correlated with the presence of pili in *Myxococcus xanthus*. *Proc Natl Acad Sci U S A* **1979**, *76*, 5952-5956, doi:10.1073/pnas.76.11.5952.
2. Yang, Y.J.; Wang, Y.; Li, Z.F.; Gong, Y.; Zhang, P.; Hu, W.C.; Sheng, D.H.; Li, Y.Z. Increasing on-target cleavage efficiency for CRISPR/Cas9-induced large fragment deletion in *Myxococcus xanthus*. *Microb Cell Fact* **2017**, *16*, 1-15, doi:10.1186/s12934-017-0758-x.
3. Julien, B.; Kaiser, A.D.; Garza, A. Spatial control of cell differentiation in *Myxococcus xanthus*. *Proc Natl Acad Sci U S A* **2000**, *97*, 9098-9103, doi:10.1073/pnas.97.16.9098.
4. Wu, S.S.; Wu, J.; Kaiser, D. The *Myxococcus xanthus* pilT locus is required for social gliding motility although pili are still produced. *Mol Microbiol* **1997**, *23*, 109-121, doi:10.1046/j.1365-2958.1997.1791550.x.
